# Cost-effectiveness of specialised manual therapy *versus* orthopaedic care for musculoskeletal disorders: long-term follow-up and health economic model

**DOI:** 10.1177/1759720X221147751

**Published:** 2023-01-31

**Authors:** Stina Lilje, Maurits van Tulder, Anders Wykman, Emmanuel Aboagye, Ulf Persson

**Affiliations:** Unit of Intervention and Implementation Research for Worker Health, Institute of Environmental Medicine (IMM), Karolinska Institute, Alfred Nobels väg 13, 171 77 Stockholm, Sweden; Faculty Behavioral and Movement Sciences, Vrije Universiteit Amsterdam, Amsterdam, The Netherlands; Orthopedic Clinic, Helsingborg Hospital, Lund University, Lund, Sweden; Unit of Intervention and Implementation Research for Worker Health, Institute of Environmental Medicine (IMM), Karolinska Institute, Stockholm, Sweden; Swedish Institute for Health Economics, Lund University, Lund, Sweden

**Keywords:** cost-effectiveness, health economic model, manual therapy, orthopaedic outpatients, quality-adjusted life years

## Abstract

**Background:**

Physiotherapy is usually the first line of treatment for musculoskeletal disorders. If pain persists, an appointment with an orthopaedic surgeon is indicated, but many disorders for which patients are placed on orthopaedic waiting lists cannot be treated in an orthopaedic clinic. Specialised manual therapy, although not mainstream, can be an effective alternative to orthopaedic care, although its cost-effectiveness beyond 12 months is unknown.

**Objectives:**

To perform an 8-year follow-up of the quality of life and costs of specialised manual therapy versus standard orthopaedic care for working-age patients with common nonsurgical musculoskeletal disorders referred to orthopaedic surgeons and to develop a health economic model.

**Design:**

Cost-effectiveness study using Markov modelling.

**Methods:**

The index group of a previously published pragmatic randomised controlled trial received a maximum of five treatment sessions of specialised manual therapy, while the control group received orthopaedic ‘care as usual’. At 3, 6, 12 and 96 months, Health-Related Quality of Life and costs were measured with Short Form Health Survey 36, Short Form Health Survey 6D and Diagnostic Related Groups. An incremental cost-effectiveness ratio was calculated, a Markov model was developed and a sensitivity analysis was performed.

**Results:**

Overall, 95% (*n* = 75) of the participants completed the 8-year follow-up. Recovery rates during the first 3 months (‘per protocol’) in the index and control group were 69% and 58%, respectively. The index group had 0.159 more gains in quality-adjusted life years and cost 40,270 SEK (€4027) less per patient over 8 years. The sensitivity analysis results were consistent with the main results.

**Conclusion:**

Specialised manual therapy dominated standard care after 8 years. The results of this small but very first study are promising; therefore, further exploration within other health care professions, clinics and/or countries is required. Our study raises questions about the triaging of orthopaedic outpatients, cost-effectiveness and resource allocation.

**Registration:**

Not applicable per the information provided by ClinicalTrials.gov.

**Plain Language Summary:**

Specialised manual therapy is more cost-effective than ‘care as usual’ for working-age patients referred to an orthopaedist. This study provides an 8-year follow-up of the cost effects and quality of life of a previously published trial.

**Why was this study conducted?**

The standard care for musculoskeletal pain consists of exercises with a physiotherapist in primary care. If the pain persists, a referral to an orthopaedic clinic is often made. Many of these referrals are inappropriate because they concern pain from muscles and joints that do not benefit from surgery or the resources available in an orthopaedic clinic. There is a gap in competence and treatment between primary and specialised care that is costly, time- and resource-consuming and causes prolonged patient suffering. Although specialised manual therapy (MT) is effective, its use is not mainstream. Costs and effects after more than 12 months of treatment that may shorten waiting lists have never been evaluated.

**What did the researchers do?**

Quality of life and costs were compared in 75 patients with nonsurgical disorders referred to orthopaedic surgeons at 8 years after treatment with specialised MT or standard orthopaedic care. A health economics model for the probability of recovery was also developed and tested.

**What did the researchers find?**

Compared with the control group, the study participants treated with specialised MT had a better quality of life, required fewer health care interventions, underwent less surgery, incurred significantly lower costs and demonstrated an increased probability of recovery.

**What do these findings mean?**

It seems probable that using specialised MT for an old, well-known structural problem may yield better treatment effects at a significantly lower cost. Our study findings suggest that policy recommendations should focus on costs and effects rather than resource utilisation alone. The study is small and requires expansion using its economic health model.

## Introduction

A considerable proportion of all appointments in primary care concern musculoskeletal pain, for which the most common interventions include advice from a general practitioner, medication, physiotherapy and/or referral for an orthopaedic consultation. Waiting lists for secondary care are often long, and earlier research has shown that many patients referred for orthopaedic consultations do not require surgery or the resources available in an orthopaedic clinic, such as surgery, advanced radiography, nerve blockades, electromyography and orthoses.^1,2^ Nevertheless, several interventions are often provided to referred patients despite not necessarily being appropriate, which is costly.^3^ Due to a lack of economic evidence and heterogeneous studies, no strong conclusions can be made about the economic effects of traditional or alternative interventions for low back and neck pain,^4^ and evidence is low regarding the cost-effectiveness of treatment of other musculoskeletal disorders in traditional health care.^5^ Specialised manual therapy [specialised manual therapy (MT); i.e. professions with a 5-year specialisation in manual treatment techniques such as osteopathy, chiropractic, naprapathy or a complete orthopaedic manual therapy (OMT) education] is an alternative to traditional health care. Naprapaths specialise in manual therapy, are licenced by the Swedish National Board on Health and Welfare and are common in Nordic countries. Their treatment involves a combination of manual techniques, such as spinal manipulation and mobilisation, and soft tissue techniques in combination with home exercises. There is clinical and scientific evidence from certain studies that specialised MT effectively treats back and neck pain^6^ and other kinds of musculoskeletal disorders in secondary care^7^ after 12 months, and a pragmatic randomised controlled trial (RCT) and health economic evaluation of naprapathic manual therapy (NMT)^8^ for nonsurgical orthopaedic outpatients showed that it dominated ‘standard care’ (i.e. better outcomes at lower cost).^9,10^ An 8-year follow-up of the same sample showed lower health care utilisation and higher quality of life (QoL) among patients who initially received specialised MT.^11^ Providing evidence for health policy decisions is valuable for both individuals and society; thus, it is important to identify evidence-based and cost-effective alternatives that may help shorten waiting lists for specialised care. Therefore, cost-effectiveness studies with follow-up periods longer than 12 months are warranted. Several similar trials are required to increase the evidence of a new treatment, which is both resource- and time-intensive. Therefore, it is convenient to create a health economic model that enables following study participants’ health states over time, altering health professionals and evaluating interventions provided by different clinics and/or countries. This study aimed to evaluate the cost-effectiveness of specialised MT in the area of NMT at 8 years after inclusion in the original trial and develop and validate a health economic model. We hypothesised that intergroup differences in the original study would remain.

## Materials and methods

### Target population, setting and treatment

**Figure 1.**
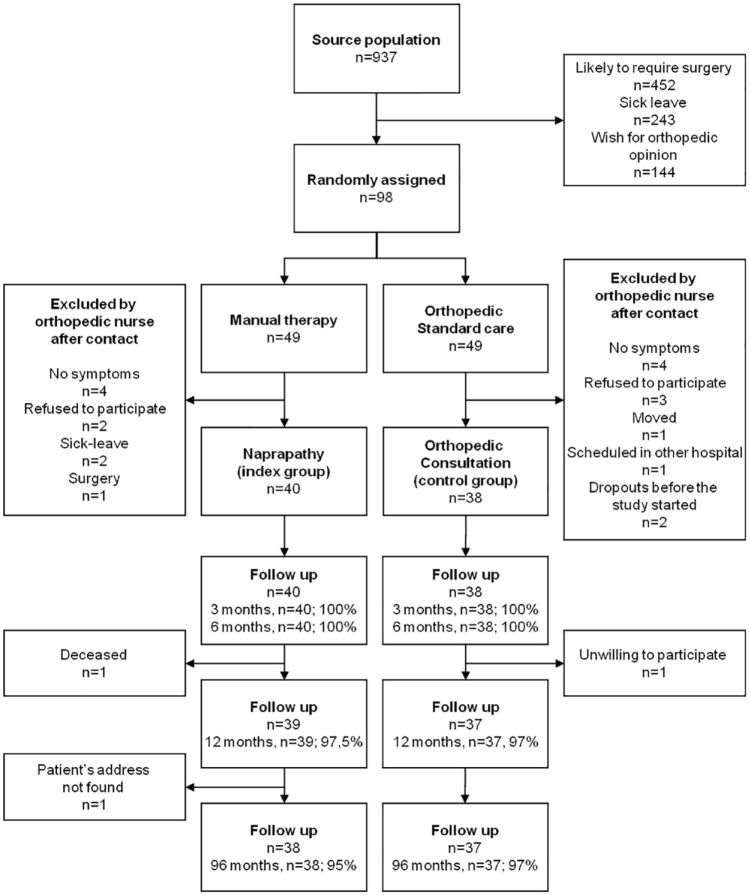
Flow chart describing the progress of the participants throughout the trial of an 8-year follow-up of quality of life and costs for working-age nonsurgical orthopaedic outpatients.

This trial was performed using ‘real-world data’ from orthopaedic outpatients of working age (*N* = 75; mean age, 42 years; 51% women) in a clinic of a county hospital in Blekinge, southern Sweden, included in a previously published pragmatic RCT and a health economic evaluation.^9,10^ The inclusion criteria (per the referral) were nonpathological and nonsurgical musculoskeletal conditions among working-age patients (Figure 1 and Table 1). The trial was performed between 1 January and 31 December 2007. The patients in the index group attended a maximum of five NMT treatment sessions consisting of different manual techniques, such as massage, stretching, myofascial trigger point treatment, mobilisation and manipulation, combined with individually tailored home exercises. The treatments in the control group were ‘care as usual’, with as many appointments and interventions as needed. The treatments in both groups were performed as usual, and the orthopaedists were unaware of which patients were included in the study. In this study, specialised MT was defined as treatments performed by professionals with a 5-year education, i.e., licenced naprapaths, chiropractors, osteopaths and physiotherapists or physicians with a complete OMT education (i.e. step 3).

**Table 1.** Diagnostic codes (International Classification of Diseases, 10th edition) documented by a naprapath or orthopaedist at the first visit.

Location	Index	Control
Neck M530, M531, M542	1	2
Shoulder/arm M190, M191B, M244 C, M294B, M653, M750, M751, M754, M770/771, M796B, S435, G560, G562C	13	11
Back M544, M545, M549, M626, Z039	5	7
Pelvis/hip M244	2	-
Knee M171, M222, M255, M626, M705, S837, Z039	5	7
Leg/foot M626, M628, M768/769, M201, M214, M242 H, G576 M722, M766, M773, M775, M796H	14	11
Summary	**40**	**38**

### Choice of health outcomes and time horizon

Health-related QoL was measured using Short Form questionnaire 36 (SF36)^12^ at 3, 6, 12 and 96 months after baseline that was mailed with the health care questionnaires. An orthopaedic nurse had telephone contact with the participants to avoid misunderstanding of the questions regarding paramedical treatments, specify which disorder to focus on and minimise loss to follow-up. All data for this study concerned only the original disorder for which the participants were referred to the orthopaedic department in the original research and for which data were collected consecutively during 2015. The study was conducted according to Consolidated Health Economic Evaluation Reporting Standards (CHEERS) statement.^13^

### Intention to treat

**Figure 2.**
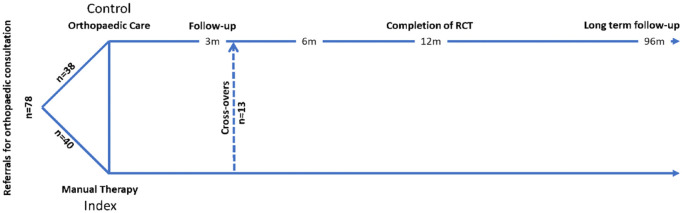
Matrix of a 96-month follow-up of a pragmatic RCT of nonsurgical orthopaedic outpatients.

For ethical reasons, the participants were allowed to cross over from the index group to the control group (i.e. orthopaedic consultation). This was performed if the patient’s disorder was thought to benefit from surgery, to elicit a second opinion, or if manual treatment had been unsuccessful. This crossover was not made until after the first ‘per protocol’ part of the study, i.e., after the 3-month follow-up (Figure 2). To prevent intruding too much on the study design, the participants in the control group continued treatment (‘care as usual’) and were not allowed to cross over.

### Estimating health outcomes and costs

The SF36 (health outcomes) was encoded to SF6D to derive QoL values used to calculate health gains and the area under the curve as quality-adjusted life years (QALYs).^14^ Data on health care consumption (costs) were collected using questionnaires containing one page for each year starting from the 12-month follow-up of the original research. The costs for health care provided within the national health care system were estimated using diagnosis-related groups^15^ and cross-checked in the hospital’s documentation system. All costs for paramedical treatments chosen by the patients were gathered from appropriate organisations. QALYs and costs were calculated as the mean values per participant in both groups when calculating the incremental cost-effectiveness ratio. In the model, costs were calculated as the mean cost per nonrecovered participant in each group at each follow-up. A health care perspective was used in which only direct costs were considered, i.e., patients who did not have any treatment at the different follow-ups were considered recovered regardless of symptom status since they did not generate any costs.

### Choice of model

A Markov model was considered appropriate for this study because it compares the probabilities of recovery of a new treatment to standard care in patients whose conditions (‘states’) change over time.^16,17^ The model comprises utilities (QoLs), costs and probabilities of recovery or recurrence. At each follow-up, participants are identified as being in one of two health states (‘recovered’ or ‘non-recovered’) or in a transition state (from ‘non-recovered’ to ‘recovered’ or from ‘recovered’ to ‘non-recovered’). From 4 months, all values were prepared for the model, i.e. the mean QoL values for columns 1–4 and 5–6, respectively, were weighed for the groups together at each follow-up and divided into ‘not recovered’ and ‘recovered’, respectively. The numbers are listed in Table 2. All values in the Markov model were calculated using Microsoft Excel.

**Table 2.** Number of participants and mean QoL values given for different treatments or no treatments for the groups from baseline to 96 months.

Months	Group	1. Specialised MT		2. Physiotherapy		3. Orthopaedics		4. Surgery		5. Recovered		6. Not recovered without treatment	
		QoL	*n*	QoL	*n*	QoL	*n*	QoL	*n*	QoL	*n*	QoL	*n*
3	Control	–	–	0.651	13	0.719	20	0.713	5	–	–	–	–
3	SMT	0.737	40	–	–	–	–	–	–	–	–	–	–
6	Control	–	–	0.650	14	0.796	2	–	–	0.791	13	0.728	9
6	SMT	–	–	–	–	0.699	10	0.537	1	0.804	24	0.616	5
12	Control	–	–	0.643	10	0.595	1	0.678	2	0.842	12	0.681	12
12	SMT	–	–	0.606	2	–	–	–	–	0.812	29	0.586	7
96	Control	–	–	0.690	10	–	–	0.568	2	0.763	20	0.615	5
96	SMT	0.808	2	0.887	1	–	–	0.785	5	0.853	26	0.649	4
QoL; SMT						SMT						Control group	
RCT baseline to 3 months						0.737						0.695	
Nonrecovered in 4–96 months						0.672						0.672	
Recovered in 4–96 months						0.811						0.811	

MT, manual therapy; QoL, quality of life; RCT, randomised controlled trial; SMT, specialised manual therapy.

The total numbers of participants are summarised horizontally.

Columns 1–4 include participants who received continuing treatment (i.e. ‘non-recovered’), while columns 5–6 include participants without treatment (i.e. ‘recovered’). The participants in column 5 recovered (i.e. were discharged from the waiting lists during the original RCT or had received treatment by the time the RCT ended but ceased to have any treatment at different times during the long-term follow-up). The participants in column 6 were considered recovered even though they still had pain/disorders, due to the health care perspective that was applied in the study.

Beneath Table 2 are QoL values prepared for the model after the ‘per protocol’ part of the trial [i.e. weighed mean values for the groups together at 4–96 months divided into ‘not recovered’ (columns 1–4) and ‘recovered’ (columns 5–6)]. ‘Not recovered’ (columns 1–4) includes patients who received treatment at any time after the ‘per protocol’ part of the trial (i.e. from 4 months onward). ‘Recovered’ includes participants who did not receive any treatment (columns 5 and 6).

### Discounting

The price level was set to 2015. Both costs and effects were discounted to supplement the base case with estimates at a common rate of 3% according to national guidelines.

### Statistics, model inputs

The individual mean QoL values were lower in the index *versus* control group at the baseline examinations in the original research; to avoid bias, this difference was adjusted for when calculating the QALY gains.^10^ Data from the participants who withdrew from the trial were used until the time of withdrawal. An intention-to-treat analysis was performed, and the participants were analysed in the group to which they were originally allocated. No imputation of missing values was performed because there were almost no missing data.

#### Utilities

The QoL values for the groups were prepared for the model; i.e., they were weighted according to the number of persons in the ‘non-recovered’ *versus* ‘recovered’ categories. The values for both groups were summarised, and a mean QoL value per participant was thereafter calculated after the ‘per protocol’ period of the study (i.e. 4–96 months; Table 2).

#### Costs

In the model, the costs from baseline to 3 months were calculated as the mean cost per patient in each group since all were considered nonrecovered by that time. Thereafter, the costs incurred at 4–6, 7–12 and 13–96 months were calculated as individual mean costs per nonrecovered patient in each group. All costs in the model were calculated for 3-month periods and are given in the Swedish SEK. Ten SEK equals approximately 1 EUR. Only the costs of surgery linked to the participants’ initial disorders were counted.

#### Probabilities

A patient’s probability of transitioning from nonrecovered to recovered from the ‘per protocol’ period was extrapolated by the model. The participants’ real-world probability of a transition from nonrecovered to recovered and vice versa was calculated by dividing the number of recovered patients in each group by the number of nonrecovered participants (and vice versa) in the previous period. These data were calculated manually for each year and divided into 3-month periods.^18^

#### Validation

A validation of the probability of recovery was validated by varying the input data and comparing the observed frequencies of real-world recovered patients with the frequency of recovered patients estimated by the model.

#### Sensitivity analysis

A univariate sensitivity analysis was performed by excluding the costs for individuals that exceeded two standard deviations of the mean individual costs for the groups.

### Ethics

The study conformed to the principles embodied by the Declaration of Helsinki, and all participants provided informed consent prior to receiving treatment. Approval was provided by the Ethical Committee in Lund, Sweden (H4 514/2006).

## Results

Ninety-five percent (*n* = 75) of participants completed the 96-month follow-up period (Figure 1). The index group had 0.159 more QALY gains (discounted value: 0.143), 40,270 lower SEK costs (€4027) (value in 2021 = 44,105 SEK) and discounted value of SEK 36,808 (€3681) per patient than those of the control group over the 8-year period. Thus, the results were dominant.

### Utilities

For the protocol-driven period (i.e. the first 3 months of the trial), the mean QoL values were 0.737 for all participants in the index group and 0.695 for the control group. For the rest of the trial (4–96 months), the QoL values were 0.811 and 0.672, respectively, for all recovered and nonrecovered participants in both groups, respectively (Table 2).

### Costs

**Figure 3.**
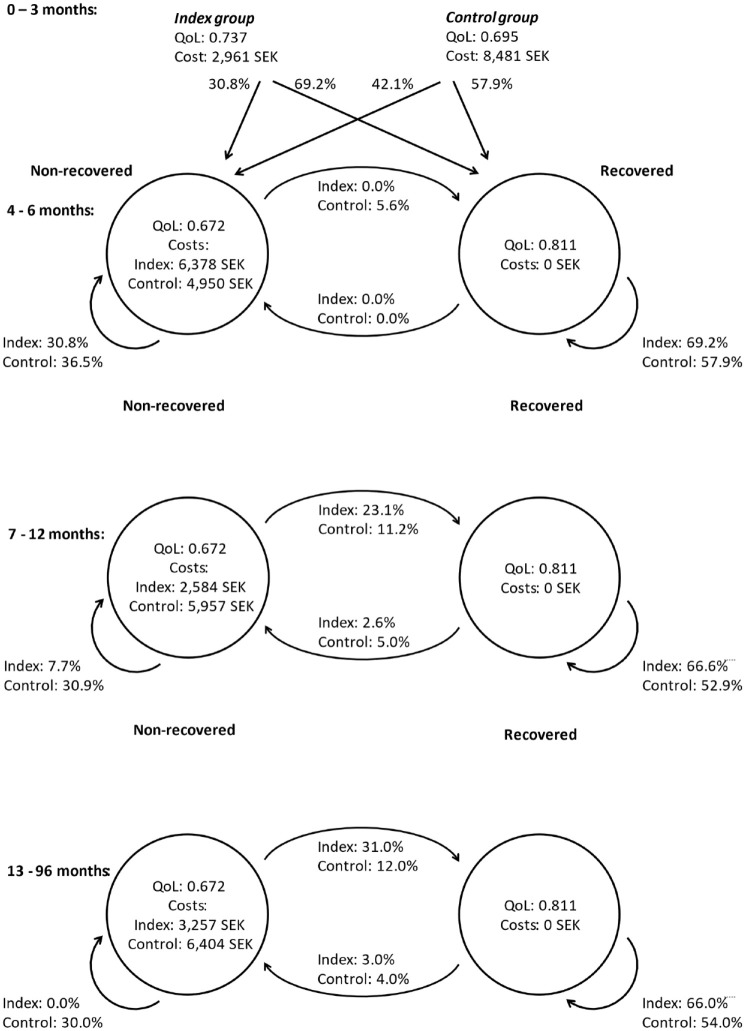
Participants’ transitions in the Markov model throughout the trial. Quality of life (QoL) scores were measured per protocol (baseline to 3 months) for each group, and for the groups together for recovered and nonrecovered patients at 4–96 months, costs were calculated for each nonrecovered patient in each group (3-month intervals), and the probabilities for recovery, nonrecovery and the two transition states were estimated at each follow-up. The short arrows that accompany the circles indicate the probability of staying in an actual condition (i.e. recovered or not recovered), while the longer arrows indicate the probability of transcending between the two states.

The total mean cost per participant from baseline to 96 months was 9003 SEK (5723–12,973) for the index group (*n* = 38) and 49,273 SEK (19,459–89,101) for the control group (*n* = 37). The mean cost per nonrecovered patient for each group is shown in Figure 3. All costs for the different treatments are presented in Table 3. The surgical procedures were as follows: diagnostic arthroscopy of the shoulder (*n* = 2), partial meniscal excision of the knee (*n* = 2), subacromial decompression of the shoulder (*n* = 1), ligamental replantation of the shoulder (*n* = 1) and chiselling of the calcaneus (*n* = 1).

**Table 3.** Costs for different interventions from baseline to 12 months and 13–96 months.

Type of intervention	Total costs for interventions, baseline to 12 months		Total costs for interventions, 13–96 months	
	Control group (*n* = 38)	Index group (*n* = 40)	Control group (*n* = 37)	Index group (*n* = 38)
Specialised MT	NA	104,580 (40)	8190 (2)	2520 (2)
Orthopaedics	106,000 (38)	30,000 (15)	17,936 (4)	–
Physiotherapy	178,596 (13)	22,878 (2)	853,392 (11)	23,184 (3)
Orthotics	1650 (6)	630 (1)	13,248 (1)	1104 (1)
Radiography/tests	37,346 (19)	19,197 (6)	–	–
Surgical procedures	187,439 (7)	16,340 (1)	72,188 (2)	114,890 (5)
Drugs/injections	6933 (18)	3141 (3)	–	–
Paramedical treatments (chiropractic, massage, lymphatic massage, personal training, shock wave)	20,790 (5)	20,054 (5)	312,074 (7)	1600 (1)
Total:	538,754 (38)	216,820 (40)	1,277,028 (37)	143,298 (38)

MT, manual therapy; NA, not applicable.

The costs are given in Swedish crowns (SEK). Ten SEK equals approximately 1 EUR.

Numbers in the parentheses represent the number of patients who received the intervention. The costs for baseline to 12 months were published previously.^8^

### Probabilities

During the first 3 months, 69% of the participants in the index group and 58% of those in the control group recovered. The probabilities for different health states varied between groups throughout the trial (Figure 3).

### Validation

**Figure 4.**
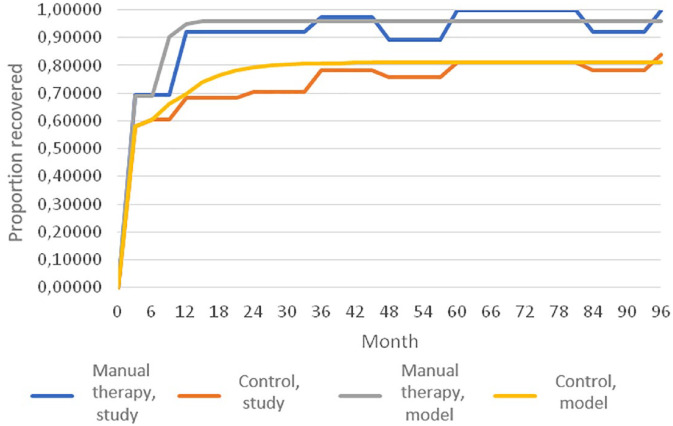
Validation of the model showing the percentage of recovered patients in a real-world follow-up study over 96 months compared with model estimation for patients treated with specialised manual therapy or standard orthopaedic care.

The probability of recovery for real-world patients at each follow-up compared with the model estimation for both groups was valid (Figure 4).

### Sensitivity analysis

When the costs for two participants in the control group that exceeded two standard deviations of the mean cost based on the total costs in both groups were removed, the individual mean cost in the control group decreased from 49,273 SEK [95% confidence interval (CI): 19,459–89,101] to 33,994 SEK (95% CI: 18,534–52,028), but it remained significantly higher than that in the index group. The value for the index group was 9003 SEK (95% CI: 5723–12,973) before and after the sensitivity analysis.

## Discussion

The QoL was higher and the costs lower for working-age nonsurgical orthopaedic patients who received specialised MT compared with standard care at 8 years after inclusion in the original RCT, thus the result is dominant. The result is mainly explained by two value drivers: a higher, faster and more continuous recovery rate from specialised MT than from standard care, demonstrated in the underlying RCT, and lower health care costs for patients treated with specialised MT.

### Specialised MT and physiotherapy

In Sweden, standard care for nonsurgical orthopaedic outpatients mostly consists of physiotherapy. What is salient with the results of our study is that general physiotherapy (i.e. no specialisation in OMT or similar) was the most common and expensive intervention in the control group at both 12 and 96 months. General physiotherapists in Sweden complete a broad 3-year education programme aimed at rehabilitating many different kinds of disorders through physical exercise.^19^ Specialised manual therapists complete a 5-year education in ‘hands-on-treatment’, i.e., manual therapy aimed at correcting joint and connective tissue dysfunction. At the time of data collection in this study, approximately 2% of all physiotherapists in Sweden were educated in specialised MT.^20^ Those who are specialised normally work in private care settings within larger cities in Sweden; thus, specialised MT is not mainstream in health care today. Hence, for most patients, the initiative to pursue specialised MT and its costs remain with them. Forty-three percent of the entire study population had already received general physiotherapy in primary care before a referral to secondary care had been made; hence, rehabilitation through exercises was presumably not the most appropriate intervention for them. General physiotherapy (i.e. first and foremost exercise) was provided for one-third of the participants in the control group; however, additional physiotherapy sessions resulted in higher costs, not better outcomes. It seems plausible that patients with musculoskeletal disorders would benefit from being triaged for the most appropriate intervention (i.e. orthopaedics, general physiotherapy or specialised MT) from the start.

### Strengths and weaknesses

To our knowledge, this is the first study with a follow-up exceeding 12 months of the cost-effectiveness of specialised MT *versus* standard care for working-age patients with the most common nonsurgical musculoskeletal disorders referred to orthopaedic surgeons. Our research question is highly relevant because musculoskeletal disorders are among the most common reasons for seeking primary health care^21^ and orthopaedic waiting lists are among the longest. Moreover, it is an internationally well-known problem that many patients referred to orthopaedic surgeons do not require surgery or the overall competence of an orthopaedic outpatient department.^1–3^ RCTs that compare a new treatment with ‘standard care’ with long-term follow-up and cost-effectiveness studies alongside are preferred by the Swedish Health Technology Assessment organisation SBU since it increases evidence-based treatment. The strengths of our study are that the model is based on an initial RCT with real-world data of longer than 12 months, and it is the first cost-effectiveness study in this field to validate a health economic model. The sample of patients/study participants mirrors orthopaedic outpatients in general with regard to age, sex, pain location and wait time,^10^ which is important for the study’s external validity. The index group yielded consistent, even increasing, statistically significant improvements at all follow-up points, which has clinical relevance. The dropout rate of the underlying trial was very low, indicating the importance of its internal validity.

Our study also has limitations that require consideration. The sample size was small, which may have decreased the precision and strength of the evidence, and therefore a power calculation for the primary outcomes of pain and physical function was performed in advance.^8^ The intention was to include patients who were considered nonsurgical, although some were finally assessed as surgical. This was due to the content of the referrals, for which appropriate tests, radiography and diagnostic competence were often lacking. In addition, the number of patients who underwent surgery for their original disorder was higher in the index group at 12–96 months (*n* = 5 in the index group, *n* = 2 in the control group); however, a total of six participants in the index group and nine in the control group had undergone surgery by the 8-year follow-up point. Another weakness is that there were no measurements between 12 and 96 months, which indicates that there might be a risk of recall bias,^22^ though the risk would be the same in both groups; hence, the long-term estimate of cost-effectiveness would not become less certain. We did not measure the compliance of the participants who were referred for physiotherapy, which is a weakness, although it was beyond the scope of this study. However, all information about the interventions within the health care system was cross-checked in the hospital’s documentation system, which resulted in a few added and subtracted physiotherapy and orthopaedic appointments in each group, thereby minimising the risk of bias. The extrapolation of the results of the first 3 months in the Markov model may have overestimated our results, which is why the treatments between baseline and 3 months were performed per protocol and ‘clean’ (i.e. only NMT *versus* orthopaedic standard care), and information about relapses was included both in the real-world follow-up and in the model. Health care consumption was also measured for each year of the follow-up period, which is why we consider the risk of overestimation as small.

### Earlier research

To the best of our knowledge, there are no studies for direct comparison. A similar design was used in a small pragmatic trial in which patients with musculoskeletal disorders consulted physiotherapists or general practitioners in primary care. It was concluded that triaging physiotherapists lead to at least as positive health effects as assessment by a general practitioner^23^ and has a high likelihood of being cost-effective, although the study sample was largely underpowered.^24^ Research on specialised MT in the shape of NMT in Sweden with up to 12 months of follow-up proved effective for patients with low back and neck pain,^6^ which is in line with studies of the costs and effects of specialised MT performed by physiotherapists and chiropractors in the Netherlands.^25^ Regarding orthopaedic outpatients, specialised MT was dominant in the original research with 12 months of follow-up,^8^ and positive short-term effects (3 months) were found when used for gatekeeping.^26^ A telephone triage of alternative management options for orthopaedic outpatients made by physiotherapists appeared cost-effective,^27^ and another trial on triage assessment of musculoskeletal disorders in orthopaedic outpatients found differences in the first few months but not at 12 months.^28^ QALYs and health care costs were estimated using Markov modelling to evaluate a physiotherapist-led service for outpatients with low back, knee or shoulder pain compared with usual orthopaedic care, and it proved that the physiotherapist-led service costs more per QALY gained but was still considered cost-effective.^29^ The diagnostic competence, assessment and triaging of patients with musculoskeletal disorders similar to those in our study performed by physiotherapists with additional postgraduate competence were evaluated, whereas the clinical outcomes of general physiotherapy (i.e. standard care) were not.

### Implications for practice

Our study showed that offering specialised MT is cost-effective compared with standard care. It is imperative that health care decision makers use evidence from cost-effectiveness studies when making decisions about limited health care resources and that the focus when treating nonsurgical orthopaedic outpatients is on cost-effectiveness rather than resource allocation. In the UK (NICE) and the Netherlands (Netherlands Healthcare Institute), e.g., it is mandatory for new interventions to be proven effective and cost-effective to be included in the public health system. Earlier research demonstrated that combining one or more manual treatment techniques with home exercises is effective,^30^ and it is important to leverage the benefits of implementing specialised MT when triaging patients with musculoskeletal disorders in a national health care system. Performing an RCT is both costly and time-consuming, which is why health economic modelling is a convenient alternative. If the profession that performs the intervention to be compared with standard care is important, then Markov modelling facilitates time- and resource-saving cost-effectiveness evaluations since it enables the possibility of changing the professions in the model. It also enables comparisons among different hospitals, clinics and countries. However, a Markov model must be fed valid and reliable data from high-quality studies.

## Conclusion

We found that specialised MT for working-age patients with common nonsurgical musculoskeletal disorders referred to orthopaedics-dominated standard care after 8 years. This very first Markov model of new competence for an old problem is promising since the effects of MT are continuously cost-effective and offer an effective and less costly alternative for treating common musculoskeletal disorders that do not require orthopaedic surgery. The study was small and requires further exploration in other health care professions, clinics and countries.

## Declarations

**Ethics approval and consent to participate:** The study conformed to the principles of the Declaration of Helsinki, and approval was granted by the Ethical Committee in Lund, Sweden (H4 514/2006). All participants provided written informed consent before the study commenced.

**Consent for publication:** Not applicable.

**Availability of data and materials:** Data may be shared upon reasonable request.
